# Co-Occurrence of Nuclear-Catenin and H3K27me3 Expression in Advanced Colorectal Cancer: A Retrospective Observational Study

**DOI:** 10.3390/curroncol33040210

**Published:** 2026-04-08

**Authors:** Ramona Abrudan, Luca Abrudan, Andreea Cămărășan, Ovidiu Camarasan, Corina Florica Ioniță, Luca Vilceanu, Ovidiu Laurean Pop

**Affiliations:** 1 Departemnt of Psychoneurosciences and Rehabilitation, Medicine and Pharmacy Faculty, University of Oradea, 410087 Oradea, Romania; 2 Radiation Oncology, Radiotherapy Laboratory, County Clinical Emergency Hospital Bihor, 410087 Oradea, Romania; luk_ali@yahoo.com; 3 Department of Morphological Disciplines, Medicine and Pharmacy Faculty, University of Oradea, 410087 Oradea, Romania; drovipop@yahoo.com; 4 Prof. Dr. Ioan Pușcaș Hospital Șimleu Silvaniei, 455300 Șimleu Silvaniei, Romania; o.camarasan@yahoo.com; 5 Department of Oncology, Topmed Hospital, 540156 Târgu Mureș, Romania; muresan.corina@gmail.com; 6 Faculty of Medicine, University of Oradea, 410087 Oradea, Romania; luca_vilceanu@yahoo.com

**Keywords:** colorectal cancer, Wnt/β-catenin pathway, H3K27me3, epigenetic

## Abstract

Colorectal cancer is a heterogeneous malignancy characterized by alterations in oncogenic signaling pathways and epigenetic mechanisms involved in gene regulation. Aberrant activation of the Wnt/β-catenin pathway represents a central molecular event in colorectal tumorigenesis, while histone-associated epigenetic modifications may contribute to tumor progression and variability. This study aimed to investigate the relationship between Wnt pathway activation and histone H3 lysine 27 trimethylation in colorectal cancer and to examine their associations with clinicopathological and molecular characteristics. A retrospective observational study was performed on 83 colorectal adenocarcinoma cases using immunohistochemical evaluation of nuclear β-catenin and H3K27me3 expression in formalin-fixed, paraffin-embedded tumor samples, together with molecular analysis of KRAS, NRAS, and BRAF mutations and microsatellite instability status. Nuclear β-catenin expression was observed in 39.8% of cases, while H3K27me3 exhibited negative, mosaic, or diffuse nuclear staining patterns. Nuclear β-catenin expression was significantly associated with patient sex and age, whereas H3K27me3 expression patterns were significantly associated with tumor location, histological grade, disease stage, and metastatic status. These results indicate that Wnt pathway activation and H3K27me3-associated epigenetic alterations frequently coexist in colorectal cancer and support the value of integrated molecular and epigenetic assessment.

## Simple Summary

Colorectal cancer is a biologically heterogeneous disease driven by complex interactions between oncogenic signaling pathways and epigenetic mechanisms of gene regulation. Activation of the Wnt signaling pathway represents a central molecular event in colorectal tumor development and progression. Epigenetic regulation, particularly histone-associated gene repression, contributes to tumor heterogeneity and disease behavior. The present study shows that Wnt pathway activation frequently co-occurs with specific histone-related epigenetic expression patterns in colorectal cancer. These findings support a coordinated role of signaling and epigenetic mechanisms in tumor progression and provide a rationale for further molecular and translational investigations.

## 1. Introduction

Colorectal cancer represents the third most frequently diagnosed malignancy worldwide as well as in Romania, with nearly two million new cases reported globally and approximately 13,000 new cases diagnosed annually in Romania. Colorectal cancer in Romania accounts for almost 6800 deaths each year, ranking as the second leading cause of cancer-related mortality [1,2,3]. Research has focused on identifying the genetic and epigenetic changes associated with colorectal carcinogenesis.

Among the best studied oncogenic drivers are mutations that hyperactivate two central signaling cascades: the Ras–Raf–MEK–ERK and the PI3K/AKT/mTOR pathways [4,5,6,7]. These pathways regulate essential processes such as proliferation, apoptosis evasion, and metabolic adaptation. Therefore, genomic studies have reported high mutation frequencies in critical components of these pathways, with KRAS mutations identified in approximately 30–50% of colorectal cancers, BRAF mutations in 10–15%, and PIK3CA mutations in 10–20% of cases [8,9,10,11].

Microsatellite instability (MSI) is a molecular phenotype resulting from defects in the DNA mismatch repair (MMR) system, leading to the accumulation of mutations within microsatellite regions of the genome. It represents a phenotypic marker of MMR deficiency and reflects the degree of genomic instability in tumor DNA. Tumors are commonly classified as MSI-high (MSI-H), MSI-low (MSI-L), or microsatellite stable (MSS). The MMR system includes key proteins (MLH1, MSH2, MSH6, and PMS2) that maintain genomic integrity by correcting DNA replication errors. Tumors with deficient MMR (dMMR) are typically associated MSI-H, whereas proficient MMR (pMMR) tumors are generally MSS [12,13,14].

Moreover, aberrant activation of the Wnt/β-catenin pathway is a key molecular feature of colorectal cancer. The Wnt signaling pathway is divided into two major categories based on the involvement of β-catenin: the non-canonical pathway, which regulates cell polarity and cell migration through the planar cell polarity (PCP) and Wnt/Ca^2+^ pathways, and the canonical Wnt/β-catenin pathway. Under physiological conditions, β-catenin is predominantly localized in the cytoplasm, where its levels are tightly regulated by a multiprotein destruction complex composed of Axin, adenomatous polyposis coli (APC), glycogen synthase kinase-3 (GSK-3), and casein kinase 1 (CK1). Upon binding of Wnt ligands to Frizzled receptors and the low-density lipoprotein receptor-related protein 5/6 (LRP5/6) co-receptors, canonical Wnt signaling is activated. This leads to Dishevelled-mediated signaling, phosphorylation of LRP5/6, recruitment of Axin, and inhibition of the β-catenin destruction complex. As a result, β-catenin becomes stabilized, accumulates in the cytoplasm, and moves into the nucleus, where it interacts with TCF/LEF transcription factors to activate Wnt target genes. Persistent activation of this pathway supports cancer stem cell self-renewal and promotes invasion, metastasis, and resistance to therapy, thereby contributing to colorectal cancer progression [15,16,17].

Epigenetic regulation involves DNA methylation and histone modifications as the main mechanisms controlling gene expression. Histones are fundamental nuclear proteins that mediate DNA compaction, spatial organization, and functional regulation within chromatin. The nucleosome core particle is composed of histones H2A, H2B, H3, and H4, while histone H1 contributes to the stabilization of higher-order chromatin structures. These histone proteins undergo various post-translational modifications such as methylation, acetylation, and phosphorylation, which dynamically modulate chromatin architecture and determine transcriptional activity or repression [18,19].

Trimethylation of lysine 27 on histone H3 (H3K27me3) is a well-characterized epigenetic mark strongly associated with transcriptional silencing. The establishment of H3K27me3 is mediated by the PRC2, whose catalytic core contains the histone methyltransferase EZH2. EZH2 utilizes S-adenosylmethionine as a methyl donor to catalyze mono-, di-, and trimethylation of H3K27. Aberrant expression or enzymatic activity of EZH2 has been associated with pathological epigenetic repression of tumor suppressor genes and is frequently implicated in oncogenic processes, including colorectal cancer development and progression [20,21,22].

The use of human tissue samples provides superior biological and clinical relevance compared to cell line models. This approach reflects two key aspects: tumor heterogeneity and the complexity of the tumor microenvironment. Unlike in vitro systems, FFPE specimens preserve tissue architecture and context-dependent molecular features, facilitating the translation of findings into clinical practice.

This is particularly relevant for biomarkers such as β-catenin and H3K27, whose expression patterns and subcellular localization are strongly influenced by tissue organization and microenvironmental factors, making their evaluation in intact tissue specimens more reliable for assessing their biological and clinical significance [23].

### Aim and Scope

The main scope of this retrospective study is to investigate the co-occurrence of aberrant Wnt signaling activation and H3K27me3-associated epigenetic regulation in colorectal cancer. Additionally, the study aims to assess the association between nuclear β-catenin expression and key clinicopathological features, including age, sex, tumor location, histological subtype, grade, stage, and metastatic pattern. It also explores factors related to H3K27me3 expression patterns and indicators of tumor aggressiveness according to disease stage, metastatic behavior, and molecular background, including KRAS, NRAS, BRAF mutational status, and MSI status.

## 2. Materials and Methods

### 2.1. Study Design and Patient Selection

The retrospective observational study included 83 patients diagnosed with colorectal adenocarcinoma. Cases were collected consecutively from the pathology archives of the Resident Laboratory, Oradea, Romania, during a time interval between 1 January 2025 and 31 October 2025. All cases had available FFPE tumor tissue suitable for immunohistochemical analysis. Clinicopathological data, including age, sex, tumor location, histological subtype, tumor grade, stage, metastatic pattern, MSI status, and mutational status (KRAS, NRAS, BRAF), were analyzed from pathology reports and medical records.

This study was conducted in full accordance with the ethical principles outlined in the Declaration of Helsinki and was approved by the Local Ethics Commission for Clinical and Research Developmental Studies, Resident Laboratory, Oradea, Romania (approval number 11, issued on 20 December 2024).

#### 2.1.1. Inclusion Criteria

Patients were included if they had a histopathological diagnosis of colorectal adenocarcinoma and available formalin-fixed, paraffin-embedded tumor tissue suitable for immunohistochemical analysis, along with complete molecular characterization, including MSI status and KRAS, NRAS mutational analysis. Only cases with successful immunohistochemical staining for β-catenin and H3K27me3, supported by appropriate internal and external controls, and with adequate tissue preservation allowing reliable evaluation of staining patterns and H-score assessment were included.

#### 2.1.2. Exclusion Criteria

Cases were excluded if tissue fixation or paraffin embedding was inadequate or if technical failure or suboptimal immunohistochemical staining occurred, including the absence of appropriate controls. Additional exclusion criteria included tissue processing artifacts or insufficient tumor material that interfered with accurate interpretation of β-catenin or H3K27me3 expression.

### 2.2. Immunohistochemical Staining

Immunohistochemical staining for β-catenin and H3K27me3 was performed on formalin-fixed, paraffin-embedded tumor tissue sections using monoclonal antibodies. β-catenin expression was evaluated using a mouse monoclonal anti-β-catenin antibody (β-catenin-1, Dako, Glostrup, Denmark), a well-established marker for assessing Wnt pathway activation through nuclear localization of β-catenin [24,25]. Wnt signaling was considered active when nuclear hot spot β-catenin staining was identified in more than 5% of tumor cell nuclei. H3K27me3 expression was assessed using a rabbit monoclonal antibody (RBT-H3K27me3, Bio SB, Santa Barbara, CA, USA) and interpreted based on nuclear staining patterns, classified as negative, mosaic, or diffuse positive. The “Mosaic” pattern was defined by heterogeneous nuclear positivity (focal clusters), whereas the “Diffuse” pattern was characterized by uniform nuclear staining in tumor cells.

Staining procedures were performed on the Dako Omnis platform in accordance with the manufacturer’s instructions [26]. Appropriate internal and external controls were included in each staining run to ensure technical validity and reproducibility. For H3K27me3, normal colonic mucosa served as an internal negative control, and external control slides were processed in parallel; in our setting, both internal and external controls for normal colonic mucosa consistently showed negative staining, rather than weak diffuse positivity as reported in some studies. All immunohistochemical slides were independently evaluated by two experienced pathologists who were blinded to the clinical and molecular data and stratified the slides based on hot spot region in negative, mosaic and diffuse pattern.

Nuclear β-catenin immunoreactivity was assessed by estimating the percentage of tumor cells exhibiting unequivocal nuclear staining. In accordance with criteria commonly reported in the literature, a 5% cut-off was applied to define nuclear positivity, such that cases with ≥5% (or >5%) of tumor cell nuclei stained were classified as positive. This threshold has been widely used to distinguish tumors with biologically relevant activation of the Wnt/β-catenin signaling pathway from cases showing only occasional or focal nuclear staining. This cut-off ensures methodological consistency and facilitates comparability with previously published studies [27,28].

### 2.3. KRAS, NRAS, BRAF Genotyping

KRAS and NRAS genotyping was performed using a real-time polymerase chain reaction (PCR) assay, the AmoyDx^®^ KRAS/NRAS Mutations Detection Kit (Amoy Diagnostics, Xiamen, China; Cat. No. ADx KRAS/NRAS-32), which detects 32 hotspot mutations across codons 12, 13, 59, 61, 117, and 146. DNA was extracted from FFPE tumor samples using the QIAamp DNA FFPE Tissue Kit (Qiagen, Hilden, Germany; Cat. No. 56404), and amplification was carried out on an ABI 7500 Fast Real-Time PCR System (Applied Biosystems, Foster City, CA, USA) according to the manufacturer’s protocol. Mutation calls were automatically generated by the AmoyDx proprietary software (https://www.amoydiagnostics.com/, accessed on 1 October 2025) using predefined Ct thresholds [29].

### 2.4. Microsatellite Instability Analysis

MSI status was determined using a five-marker mononucleotide PCR panel (BAT-25, BAT-26, NR-21, NR-24, MONO-27; Promega MSI Analysis System v1.2) [30]. DNA was extracted from FFPE tumor tissue using the QIAamp DNA FFPE Tissue Kit (Qiagen, Cat. No. 56404), according to the manufacturer’s protocol. PCRs were prepared in a 25-μL volume with standard reagent concentrations. PCR amplification was performed with an initial denaturation at 95 °C for 10 min, followed by 35 cycles of denaturation at 95 °C for 30 s, annealing at 55 °C for 30 s, and extension at 72 °C for 30 s, with a final extension at 72 °C for 10 min. Amplicons were analyzed by capillary electrophoresis on an ABI Prism 3130xl Genetic Analyzer (Applied Biosystems), and fragment analysis was performed using GeneMapper software v5.0. Tumors were classified as MSI-H if instability was present in ≥2 loci, MSI-L if present in one locus, and MSS if no instability was observed, according to established guidelines [30,31]

### 2.5. Statistical Analysis

Statistical analyses were performed using Statistical Package of Social Science (SPSS), version 26. Categorical variables were summarized as frequencies and percentages. Associations between β-catenin expression, H3K27me3 patterns, and clinicopathological or molecular variables were evaluated using chi-square or Fisher’s exact tests, as appropriate.

Multivariate binary logistic regression models were constructed to identify independent predictors of nuclear β-catenin expression and H3K27me3 positivity (mosaic/diffuse vs. negative). Odds ratios (ORs) with 95% confidence intervals (CIs) were calculated. A *p*-value < 0.05 was considered statistically significant.

## 3. Results

The study cohort included 83 patients with colorectal adenocarcinoma, predominantly male (61.4%), with most patients aged between 51 and 70 years. Tumors were more frequently located in the left colon (71.1%) and were mainly conventional adenocarcinomas (84.3), with a predominance of moderately differentiated tumors (G2, 85.5%). Advanced-stage disease was common, with stage IV tumors representing 67.5% of cases and hepatic involvement being the most frequent metastatic site (Table 1). Nuclear β-catenin expression, indicative of active Wnt signaling, was observed in 39.8% of cases, while H3K27me3 expression showed negative or mosaic patterns in the majority of tumors. Molecular analysis demonstrated KRAS mutations in 55.4% of cases, whereas NRAS and BRAF mutations were each identified in 9.6% of tumors. Microsatellite instability testing revealed MSI-H status in 9.6% of cases, with the remaining tumors classified as microsatellite stable (MSS).

**Figure 1:**
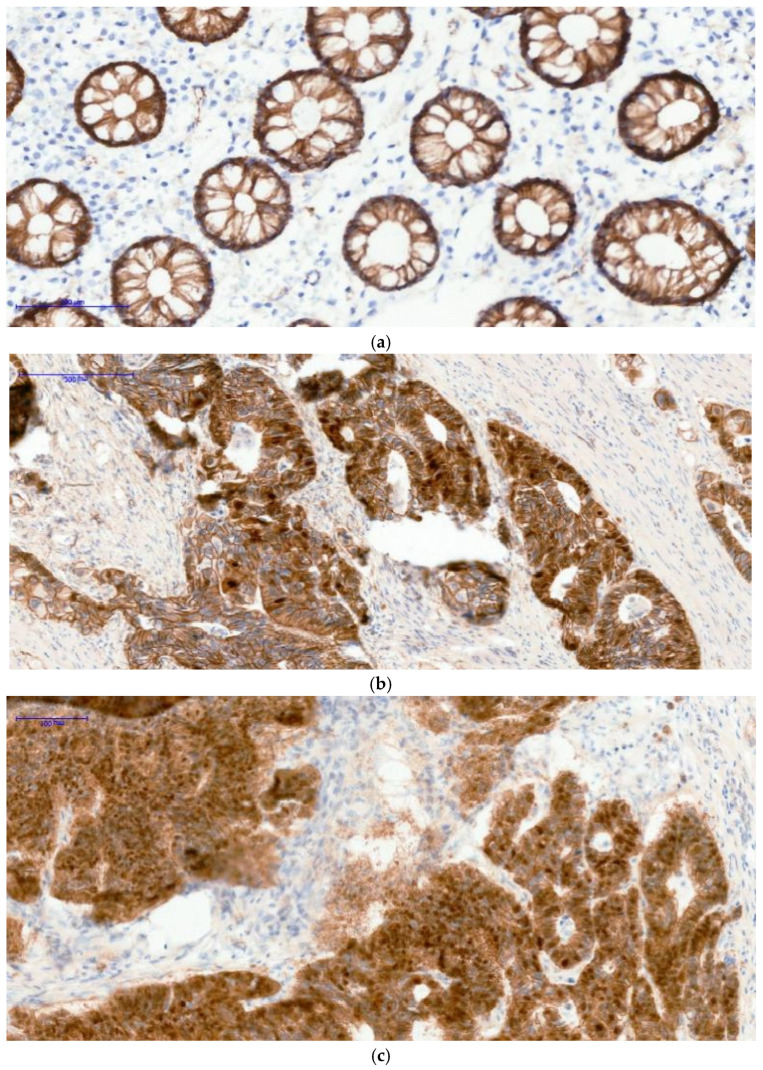
The images below illustrate external control staining for β-catenin in normal colonic mucosa, in which all epithelial gland nuclei remain negative (image (**a**)), while the cell membranes and cytoplasm show physiological β-catenin immunoreactivity. The other two images (image (**b**,**c**)) demonstrate nuclear β-catenin positivity in tumor tissue; in case C, nearly all adenocarcinoma cell nuclei exhibit strong nuclear staining. (**a**) β-catenin 20xOB-Control-negative nuclei; (**b**) β-catenin 20xOB-Positive 30% nuclei; (**c**) β-catenin 20xOB-Positive 90% nuclei. Brown staining indicates positive β-catenin expression, while blue represents nuclear counterstaining.

**Figure 2:**
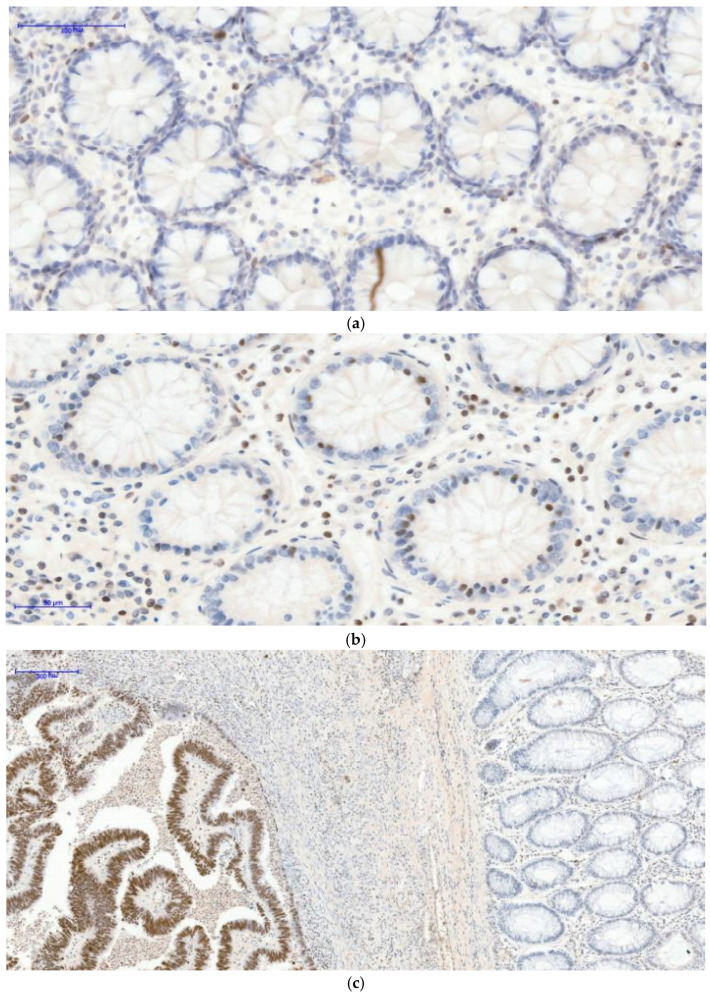
(**a**) H3K27me3 External control 20XOB-negative glandular nuclei, some positive nuclear cells of lamina propria: Based on internal and external controls for H3K27me3, we observed that the nuclei of normal glandular epithelial cells remain negative, while nuclear staining is present in cells of the lamina propria. (**b**) H3K27me3 External control 20XOB-negative glandular nuclei, more positive nuclear cells of lamina propria (inflammatory condition): A higher density of inflammatory cells with nuclear positivity is evident, an inflammatory condition of the colon. In the (**c**) H3K27me3 Internal control 10XOB-Positive tumoral nuclei (**left** side), negative epithelial nuclei normal glands (**right** side) image, serving as an internal control, a clear contrast is observed: The tumor tissue on the left side shows strong diffuse nuclear positivity, whereas the adjacent normal colonic mucosa on the right side demonstrates complete absence of nuclear staining in the glandular epithelium. Brown staining indicates positive H3K27me3 expression, while blue represents nuclear counterstaining.

Significant associations were observed between clinicopathological variables and both β-catenin expression and H3K27me3 patterns. Nuclear β-catenin expression showed significant associations with sex and age group, being more frequently observed in female and younger patients within this cohort (Table 2), (Figure 1a–c). H3K27me3 expression patterns were significantly associated with tumor location, grade, stage, and metastatic status, indicating a relationship between epigenetic alterations and advanced disease characteristics (Table 2), (Figure 2a–c). No significant associations were observed between β-catenin expression and tumor location, grade, stage, or metastatic status.

As presented below, in Table 3, a significant association was identified between nuclear β-catenin expression and H3K27me3 expression patterns, supporting a relationship between Wnt pathway activation and epigenetic regulation. Nuclear β-catenin expression was also significantly associated with MSI status and with KRAS/NRAS/BRAF mutational status. H3K27me3 expression patterns showed significant associations with gene mutation status, whereas no significant associations were observed between H3K27me3 expression and MSI status.

In multivariate binary logistic regression assessing predictors of nuclear β-catenin expression, female sex and younger age remained independently associated with nuclear/active β-catenin. Female patients had higher odds of nuclear β-catenin compared to males (OR 8.83, 95% CI 2.65–29.44; *p* = 0.0004), whereas age ≥ 60 years was associated with lower odds compared to <60 years (OR 0.20, 95% CI 0.06–0.65; *p* = 0.0077) (Table 4). Stage IV disease and the presence of metastasis showed non-significant trends toward increased odds of nuclear β-catenin; however, confidence intervals could not be reliably estimated due to sparse data in some categories.

**Figure 3:**
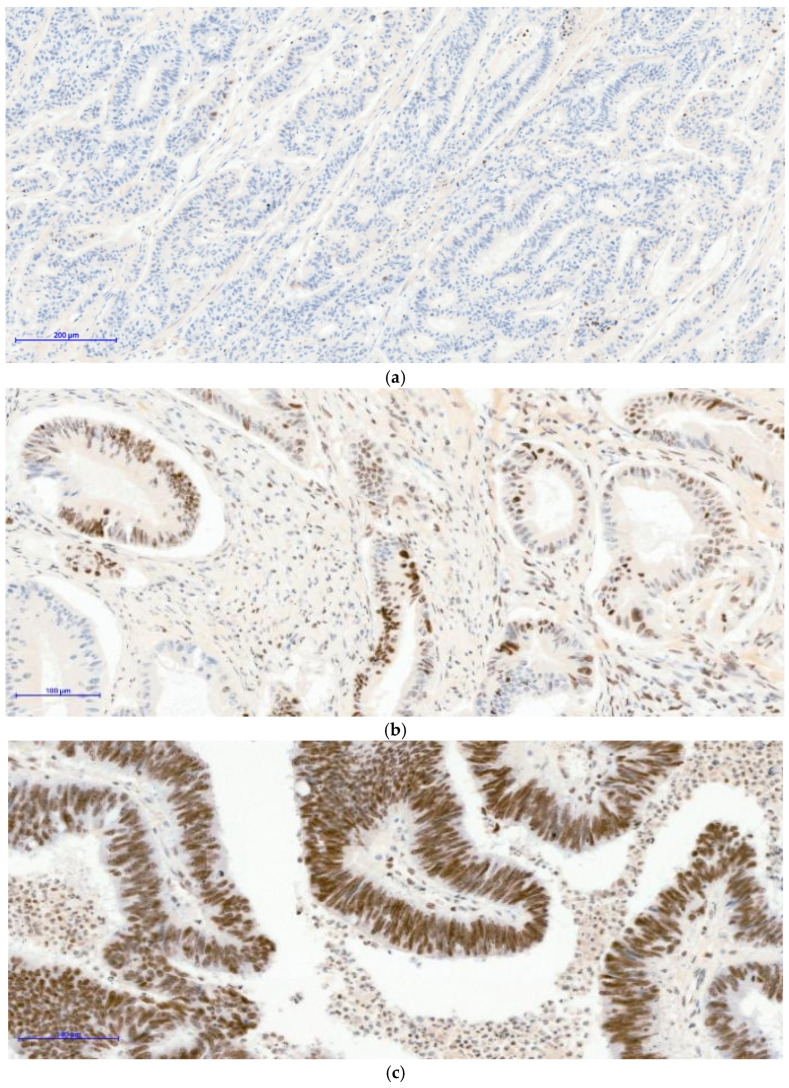
Three distinct nuclear staining patterns were observed in colorectal tumor samples: negative expression [image (**a**) H3K27me3 10XOB-negative tumoral nuclei], a mosaic pattern characterized by heterogeneous nuclear positivity [image (**b**) H3K27me3 20XOB-positive tumoral nuclei, mosaic pattern], and a strong diffuse pattern with uniform nuclear staining across tumor cells [image (**c**) H3K27me3 20XOB-positive tumoral nuclei, diffuse pattern (100%)]. Brown staining indicates positive H3K27me3 expression, while blue represents nuclear counterstaining.

In the second multivariate model evaluating predictors of H3K27me3 positivity (mosaic-Figure 3b/diffuse-Figure 3c vs. negative-Figure 3a), nuclear β-catenin was identified as an independent predictor of H3K27me3 expression (OR 4.92, 95% CI 1.24–19.55; *p* = 0.024) (Table 5); therefore, cases with nuclear β-catenin expression had approximately five-fold higher odds of exhibiting a mosaic or diffuse H3K27me3 pattern compared with negative expression. Age ≥ 60 years and female sex were not significantly associated with H3K27me3 status, while MSI-H showed a non-significant positive association with wide confidence intervals, likely reflecting limited subgroup size.

## 4. Discussion

The results of the present study should be interpreted with consideration of potential bias related to tumor grade, particularly in advanced-stage and high-grade (grade IV) colorectal carcinomas. These tumors are often underrepresented in retrospective cohorts and show increased biological and epigenetic heterogeneity, which may contribute to variability in immunohistochemical results and reduced statistical power. As a result, associations involving advanced disease stage or metastatic status may appear weaker or fail to reach statistical significance, reflecting limited subgroup size or within-group heterogeneity rather than the absence of a true biological relationship.

These limitations highlight the need for cautious interpretation of findings derived from high-grade tumors and support further validation in larger, stage-balanced cohorts. Future studies with prospective designs and more homogeneous clinical stratification may help clarify the role of Wnt/β-catenin signaling across different stages of colorectal cancer progression.

Colorectal cancer represents a biologically heterogeneous malignancy driven by intricate interactions between oncogenic signaling pathways and epigenetic regulatory mechanisms. Although aberrant activation of the Wnt/β-catenin pathway is a well-established hallmark of colorectal tumorigenesis, the contribution of histone-mediated epigenetic modifications remains comparatively underexplored. In particular, current evidence regarding histone H3 lysine 27 trimethylation (H3K27me3) in colorectal cancer is limited, with only a small number of studies examining its global distribution and intratumoral heterogeneity. This relative scarcity of data highlights the need for further systematic investigation into the crosstalk between oncogenic signaling pathways and histone-based epigenetic regulation in colorectal cancer.

Dysregulation of histone H3 lysine 27 trimethylation (H3K27me3) has been widely implicated in tumor progression and clinical prognosis across diverse malignancies. Prior studies have consistently demonstrated that reduced H3K27me3 expression is associated with adverse clinicopathological features, including increased tumor size, advanced disease stage, estrogen receptor negativity, and lymph node involvement in breast carcinoma, as well as higher tumor grade and stage in ovarian cancer and higher tumor grade in pancreatic cancer [20,32]. In concordance with these observations, the present study demonstrates a statistically significant association between advanced disease stage—reflected by increased tumor size—and altered H3K27me3 expression. This co-occurrence further supports the notion that signaling pathway activation and epigenetic alterations are closely interconnected in colorectal cancer.

Previous studies have reported heterogeneous associations between nuclear β-catenin expression and clinicopathological characteristics in colorectal cancer. Gao et al. observed significant associations between nuclear β-catenin expression and TNM stage, lymph node involvement, and histological differentiation, while no significant correlations were identified with patient age or sex [33]. Hussein et al. reported significant relationships between nuclear β-catenin expression, patient age, lymph node involvement, and distant metastases [34]. Current evidence has not identified a clear sex-specific difference in the activation of the WNT signaling pathway in colorectal cancer. Available data suggest that sex-related differences are more likely to reflect variations in the regulatory architecture of this pathway. In this context, previous studies have shown that genes involved in WNT signaling may be more strongly targeted within gene regulatory networks in male tumors, whereas in females, alternative pathways, particularly those related to metabolism, appear to be more prominently regulated. Additionally, hormonal influences, particularly estrogen, have been proposed to modulate WNT/β-catenin signaling, potentially contributing to sex-related differences in tumor behavior [35,36]. Our findings suggest a relative predominance of WNT pathway involvement in younger female patients. However, these results should be interpreted with caution, given the specific characteristics and size of our cohort, as well as the exploratory nature of the analysis.

Although advanced disease stage and metastatic status were associated with increased odds of nuclear β-catenin accumulation, these associations did not reach statistical significance. This lack of statistical significance may reflect limited sample size or reduced statistical power within specific subgroups rather than the absence of a biological relationship. Accordingly, the observed patterns indicate variability in Wnt/β-catenin pathway activity across patient subgroups and disease contexts.

In the second multivariate model, nuclear β-catenin expression emerged as the strongest independent predictor of H3K27me3 positivity, supporting a close relationship between Wnt pathway activation and epigenetic regulation. The approximately five-fold increased likelihood of mosaic or diffuse H3K27me3 expression in tumors with nuclear β-catenin suggests a potential functional interplay between oncogenic signaling and chromatin-modifying mechanisms. Beyond its statistical significance, this predictive relationship may reflect an underlying biological link, whereby activation of the Wnt/β-catenin pathway contributes to the establishment of specific epigenetic states within tumor cells. To further contextualize these associations within a biological framework, it is relevant to consider the role of Polycomb-mediated epigenetic regulation in colorectal cancer. The PRC2 represents a major chromatin-regulatory system implicated in the maintenance of tissue homeostasis. PRC2 is composed of the core subunits EZH1/2, EED, SUZ12, and RBAP46/48, with EZH2 functioning as the catalytic lysine methyltransferase responsible for H3K27me3, a modification commonly associated with chromatin compaction and transcriptional repression [37,38,39]. Although EZH2 overexpression has been frequently reported in colorectal cancer, its functional implications remain incompletely defined and appear to be context dependent. Experimental evidence suggests that activation of Wnt/β-catenin signaling may locally attenuate PRC2-mediated H3K27me3 at Wnt target gene promoters, thereby permitting transcriptional activation. In contrast, sustained β-catenin signaling has been reported to coincide with broader alterations in the genomic distribution of H3K27me3, which may reflect epigenetic reprogramming processes occurring during tumor progression rather than direct causal mechanisms [38].

Although MSI-H status was associated with a non-significant increase in H3K27me3 expression, the wide confidence intervals necessitate cautious interpretation of this observation. This finding may reflect limited statistical power and highlights the need for further evaluation in larger, well-characterized cohorts, particularly given the scarcity of existing data addressing the relationship between microsatellite instability and H3K27me3-associated epigenetic regulation.

Data regarding global H3K27me3 expression in colorectal cancer remain limited in the literature, with only a small number of studies addressing this epigenetic mark [32,40]. An early investigation by Nakazawa et al. reported no significant differences in global nuclear H3K27me3 expression among normal colorectal mucosa, adenomas, and adenocarcinomas, nor across tumor differentiation grades or histological subtypes, suggesting relative stability of this epigenetic mark at a global level [40]. In contrast, the present findings describe distinct negative, mosaic, and diffuse nuclear H3K27me3 staining patterns, indicating a degree of epigenetic heterogeneity that may not be captured by global expression assessments alone. Notably, the mosaic pattern reflects intratumoral variability in chromatin regulation and supports the concept of dynamic epigenetic remodeling during tumor progression rather than a fixed epigenetic phenotype, with diffuse positivity potentially representing a more uniform and stabilized epigenetic state across tumor cells.

The strong association between nuclear β-catenin expression and mosaic or diffuse H3K27me3 patterns observed in multivariate analysis supports the concept of a coordinated relationship between Wnt pathway activation and epigenetic regulation in colorectal cancer. Therefore, the presence of heterogeneous H3K27me3 expression patterns may contribute to variability in tumor behavior and therapeutic response, although further studies are required to clarify their prognostic or predictive significance. Taken together, the observed co-occurrence and predictive association support the concept of coordinated interaction between oncogenic signaling and epigenetic regulation in colorectal cancer progression.

Accumulating evidence supports a complex and context-dependent relationship between Wnt/β-catenin signaling and PRC2-mediated epigenetic regulation. Activation of Wnt signaling and nuclear accumulation of β-catenin have been reported to locally antagonize PRC2 activity and reduce H3K27me3 deposition at specific Wnt target gene loci; however, this effect appears to be largely locus specific rather than global in nature [41]. In parallel, several studies have suggested that sustained β-catenin activation may be associated with broader epigenetic reprogramming, including alterations in PRC2 activity and redistribution of H3K27me3 toward alternative genomic regions, such as genes involved in differentiation and tumor suppression. In line with this concept, the present findings indicate that nuclear β-catenin and H3K27me3 can coexist within the same tumor samples, and importantly, that nuclear β-catenin acts as a significant predictive factor for H3K27me3 expression [19,42]. These observations are compatible with a model in which nuclear β-catenin localization is associated with a tumor context characterized by increased epigenetic plasticity, whereby β-catenin-driven transcriptional activity may coexist with compensatory or adaptive PRC2-mediated repression at other genomic loci. In this setting, β-catenin signaling and H3K27me3-dependent chromatin regulation appear to operate in a coordinated, though not necessarily mutually exclusive, manner during colorectal cancer progression.

Among the relatively few studies exploring the interaction between epigenetic regulation and the Wnt/β-catenin signaling pathway, Serman et al. and Hoyle et al., reported a potential mechanistic link between epigenetic dysregulation and Wnt/β-catenin signaling during colorectal carcinogenesis, through two mechanism: one indirect, involving promoter hypermethylation-mediated silencing of Wnt pathway inhibitors, which removes inhibitory control and one direct histone-modifying enzymes that directly modulate chromatin accessibility at Wnt target gene promoters, thereby promoting aberrant pathway activation [43,44].

The observed association between Wnt/β-catenin activation and H3K27me3 expression may have potential therapeutic implications. Tumors characterized by active Wnt signaling and increased epigenetic plasticity may represent candidates for future strategies combining Wnt pathway inhibitors with epigenetic-targeting agents such as EZH2 inhibitors.

### Limitations of the Study

The presented study has several limitations. First, it has a retrospective design and relatively small sample size may have limited statistical power, particularly in subgroup analyses, resulting in wide confidence intervals in multivariate models. The single-population setting may also limit the generalizability of the findings. The observed associations between Wnt pathway activation and H3K27me3-associated epigenetic regulation suggest the existence of potential biological links between these processes. However, these results should be interpreted with caution, as the analyses were performed in a single laboratory, which may introduce methodological and technical limitations and affect the generalizability of the findings. The exploratory nature of the analysis, together with the variability of reported associations between nuclear β-catenin expression and clinicopathological parameters, necessitates cautious interpretation and may limit the external validity of the findings, particularly regarding age- and sex-related patterns. Prospective studies integrating immunohistochemical assessment of EZH2 may contribute to a more detailed understanding of the mechanistic relationship between PRC2-mediated epigenetic regulation, H3K27me3 deposition, and Wnt/β-catenin signaling in colorectal cancer.

This study may be limited by the underrepresentation and increased heterogeneity of advanced-stage, high-grade colorectal carcinomas, which could influence the strength of observed associations. Consequently, further validation through independent, multicenter cohorts, as well as functional and prospective studies, is required to confirm these observations and to establish a causal relationship.

## 5. Conclusions

In this study, activation of the Wnt/β-catenin pathway was frequently observed together with H3K27me3 expression in colorectal cancer. Tumors showed different H3K27me3 staining patterns, indicating that epigenetic regulation is not uniform across all tumor cells.

Nuclear β-catenin was independently associated with mosaic and diffuse H3K27me3 expression, suggesting that epigenetic changes may occur alongside Wnt pathway activation in colorectal tumors. This association may help explain differences observed between tumors and within individual tumors.

Although causal relationships cannot be established, these findings highlight the importance of evaluating epigenetic features together with key signaling pathways in colorectal cancer. Further studies are needed to better understand the biological and clinical relevance of these interactions.

These findings should be interpreted as hypothesis-generating and require validation in larger, prospective studies.

## Figures and Tables

**Table 1:** Clinicopathological, Molecular, and Immunohistochemical Characteristics of the Study Cohort (*n* = 83).

Variable	Category	n (%)
Sex	Male	51 (61.4)
	Female	32 (38.6)
Age (years)	30–40	8 (9.6)
	41–50	8 (9.6)
	51–60	23 (27.7)
	61–70	24 (28.9)
	71–80	20 (24.1)
Tumor location	Right colon	24 (28.9)
	Left colon	59 (71.1)
Histological type	Adenocarcinoma	70 (84.3)
	Mucinous adenocarcinoma	13 (15.7)
Tumor grade	G1	8 (9.6)
	G2	71 (85.5)
	G3	4 (4.8)
Tumor stage	II	4 (4.8)
	III	23 (27.7)
	IV	56 (67.5)
Metastatic status	None	27 (32.5)
	Hepatic	38 (45.8)
	Pulmonary	10 (12.0)
	Other or multiple sites	8 (9.7)
MSI status	MSS	75 (90.4)
	MSI-H	8 (9.6)
Gene mutation status	Wild-type	21 (25.3)
	KRAS	46 (55.4)
	NRAS	8 (9.6)
	BRAF	8 (9.6)
β-catenin expression	Membranous	50 (60.2)
	Nuclear (active Wnt)	33 (39.8)
H3K27me3 expression pattern	Negative	42 (50.6)
	Mosaic	33 (39.8)
	Diffuse positive	8 (9.6)

Data are presented as number (percentage). β-catenin nuclear expression was considered indicative of active Wnt signaling when more than 5% of tumor cell nuclei showed immunoreactivity. H3K27me3 expression was classified based on nuclear staining patterns as negative, mosaic, or diffuse positive. MSI- microsatellite instability.

**Table 2:** Associations between β-catenin Expression, H3K27me3 Patterns, and Clinicopathological Variables.

Clinicopathological Variable	Comparison (Chi-Square Test)	*p*-Value
Sex	β-catenin (membranous vs. nuclear)	**0.001**
Sex	H3K27me3 pattern	**0.038**
Age group	β-catenin (membranous vs. nuclear)	**<0.001**
Age group	H3K27me3 pattern	**0.007**
Tumor location	β-catenin (membranous vs. nuclear)	0.446
Tumor location	H3K27me3 pattern	**0.003**
Tumor grade	β-catenin (membranous vs. nuclear)	0.220
Tumor grade	H3K27me3 pattern	**<0.001**
Tumor stage	β-catenin (membranous vs. nuclear)	0.238
Tumor stage	H3K27me3 pattern	**<0.001**
Metastatic status	β-catenin (membranous vs. nuclear)	0.105
Metastatic status	H3K27me3 pattern	**<0.001**
Histological type	β-catenin (membranous vs. nuclear)	0.258
Histological type	H3K27me3 pattern	**0.049**

Bold values indicate statistically significant associations (*p* < 0.05). Associations were assessed using the Pearson chi-square test; Fisher’s exact test was applied when expected cell counts were <5. β-catenin nuclear expression was considered indicative of active Wnt signaling. H3K27me3 expression was classified as negative, mosaic, or diffuse positive.

**Table 3:** Associations between β-catenin Expression, H3K27me3 Patterns, and Molecular Variables.

Molecular Variable	Comparison	*p*-Value
β-catenin	H3K27me3 pattern	**0.002**
MSS status	β-catenin (membranous vs. nuclear)	**0.016**
MSS status	H3K27me3 pattern	0.581
Gene mutation status (KRAS/NRAS/BRAF)	β-catenin (membranous vs. nuclear)	**0.004**
Gene mutation status (KRAS/NRAS/BRAF)	H3K27me3 pattern	**<0.001**

Bold values indicate statistically significant associations (*p* < 0.05). Associations were assessed using the Pearson chi-square test; Fisher’s exact test was applied when expected cell counts were <5. β-catenin nuclear expression was considered indicative of active Wnt signaling. H3K27me3 expression was classified as negative, mosaic, or diffuse positive.

**Table 4:** Multivariate logistic regression for nuclear β-catenin expression.

Predictor	OR	95% CI	*p*-Value
Female sex	8.83	2.65–29.44	0.0004
Age ≥ 60 years	0.20	0.06–0.65	0.0077
Metastasis (any vs. none)	2.07	CI not reliably estimated	n.s.
Stage (IV vs. II–III)	2.53	CI not reliably estimated	n.s.

Odds ratios (ORs) were estimated using logistic regression. CI denotes the 95% confidence interval. n.s. indicates lack of statistical significance (*p* ≥ 0.05). Confidence intervals for some predictors were not reliably estimable owing to small sample size and model instability.

**Table 5:** Multivariate logistic regression for H3K27me3 positivity (mosaic/diffuse vs. negative).

Predictor	OR	95% CI	*p*-Value
Nuclear β-catenin	4.92	1.24–19.55	0.024
Age ≥ 60 years	0.43	0.10–1.75	0.237
Female sex	0.38	0.09–1.63	0.193
MSI-H	6.65	0.66–66.62	0.107

OR, odds ratio; CI, confidence interval; MSI-H, microsatellite instability-high. ORs were derived from multivariate logistic regression analysis. CI denotes the 95% confidence interval; *p*-values < 0.05 were considered statistically significant.

## Data Availability

The original contributions presented in this study are included in the article. Further inquiries can be directed to the corresponding author.

## Abbreviations

The following abbreviations are used in this manuscript:

AKT	Protein kinase B
APC	Adenomatous polyposis coli
BRAF	B-Raf proto-oncogene, serine/threonine kinase
CI	Confidence interval
CK1	Casein kinase 1
CRC	Colorectal cancer
Ct	Cycle threshold
DNA	Deoxyribonucleic acid
EED	Embryonic ectoderm development
ERK	Extracellular signal-regulated kinase
EZH1/2	Enhancer of zeste homolog 1/2
EZH2	Enhancer of zeste homolog 2
FFPE	Formalin-fixed, paraffin-embedded
FIGO	International Federation of Gynecology and Obstetrics
GSK-3	Glycogen synthase kinase 3
H3K27me3	Trimethylation of lysine 27 on histone H3
H-score	Histological score
IHC	Immunohistochemical
KRAS	Kirsten rat sarcoma viral oncogene homolog
LEF	Lymphoid enhancer-binding factor
LRP5/6	Low-density lipoprotein receptor-related protein 5/6
MEK	Mitogen-activated protein kinase kinase
MMR	Mismatch repair
MSS	Microsatellite stable
MSI	Microsatellite instability
MSI-H	Microsatellite instability-high
MSI-L	Microsatellite instability-low
mTOR	Mechanistic target of rapamycin
NRAS	Neuroblastoma RAS viral oncogene homolog
OB	Objective (microscope objective)
OR	Odds ratio
PCP	Planar cell polarity
PCR	Polymerase chain reaction
PD-L1	Programmed cell death protein 1
PI3K	Phosphoinositide 3-kinase
PRC2	Polycomb repressive complex 2
RBAP46/48	Retinoblastoma-associated proteins 46/48
RAS	Rat sarcoma virus oncogene family
SPSS	Statistical Package for the Social Sciences
SUZ12	Suppressor of zeste 12
TCF	T-cell factor
TNM	Tumor–node–metastasis staging system
Wnt	Wingless-related integration site
